# Efficacy of laparoscopic sleeve gastrectomy in obese patients with type 2 diabetes mellitus: A protocol of systematic review and meta-analysis

**DOI:** 10.1097/MD.0000000000020535

**Published:** 2020-06-05

**Authors:** Wei-wei Wei, Xian-dong Fu, De-wang Su, De-zhi Ke, Rong-rong Yao, Ke-yan Chen, Hao Tian

**Affiliations:** a Department of General Surgery; b Department of Interventional Radiology; c Department of Endocrinology, First Affiliated Hospital of Jiamusi University, Jiamusi, China.

**Keywords:** efficacy, laparoscopic sleeve gastrectomy, type 2 diabetes mellitus

## Abstract

**Background:**

A numerous studies have reported that obese patients (OP) are easily to have type 2 diabetes mellitus (T2DM). Although a variety of managements are available to treat such disorder, their efficacy is still limited. Previous studies have reported that laparoscopic sleeve gastrectomy (LSGT) can benefit OP with T2DM. However, no study specifically and systematically explores this topic. Thus, this study will assess the efficacy and complications of LSGT for the management of OP with T2DM.

**Methods:**

The search strategy will be performed in the electronic databases from inception to the March 31, 2020 without limitations of language and publication time: PUBMED, EMBASE, Cochrane Library, Scopus, Web of Science, CINAHL, AMED, WANGFANG, VIP, and CNKI. Two authors will independently identify the articles, collect the data, and assess the risk of bias using Cochrane risk of bias tool. We will invite a third author to solve any differences between two authors. We will use RevMan 5.3 software to investigate the statistical analysis.

**Results:**

This study will supply a high-quality synthesis of randomized controlled trials (RCTs) on the analysis of LSGT for the management of OP with T2DM.

**Conclusions:**

This study will help to build proposals that aim at providing high quality RCTs in the management of LSGT in OP with T2DM.

**Systematic review registration:**

INPLASY202040128.

## 1 Introduction

It is well known that obesity is one of the most common risk factors for patients with type 2 diabetes mellitus (T2DM).^[1–4]^ A huge number of studies found that T2DM has very close association with obesity.^[5–8]^ Thus, obesity management is recommended as effective strategy for the prevention and treatment of T2DM.^[9,10]^ Although a variety of managements can help to treat T2DM, their efficacy is still far from satisfaction.^[11–13]^

Published studies have reported laparoscopic sleeve gastrectomy (LSGT) can effectively mange OP with T2DM.^[14–22]^ However, its efficacy is still unclear at literature level. Thus, this systematic study will assess the efficacy and complications of LSGT for the management of OP with T2DM.

## 2 Methods

### 2.1 Study registration

The current protocol review has been registered through INPLASY202040128. It will be reported according to the Preferred Reporting Items for Systematic Reviews and Meta-Analyses Protocols guideline.^[23]^

### 2.2 Inclusion criteria for study selection

#### 2.2.1 Types of studies

This study will include randomized controlled trials (RCTs) alone that focusing on the efficacy and complications of LSGT for the management of OP with T2DM. We will exclude any other studies, such as animal studies, case reports, case series, reviews, comments, non-clinical trials, non-controlled trials, and quasi-RCTs.

#### 2.2.2 Types of participants

Any participants who were clinically diagnosed as OP with T2DM will be considered for inclusion regardless their nationality, race, sex, and age.

#### 2.2.3 Types of interventions

All participants in the experimental group received any forms of LSGT intervention.

In the control group, we will include participants who underwent any managements, but not LSGT.

#### 2.2.4 Types of outcome measurements

The primary outcome is complete remission of T2DM (defined as blood hemoglobin A1C (HbA1c) <6% (42 mmol/mol)).

The secondary outcomes are body mass index, partial remission of T2DM (defined as blood HbA1c < 6.5% (48 mmol/mol)), lipids, high sensitivity C-reactive protein, quality of life (measured as any relevant scales), and complications.

### 2.3 Search methods for the identification of studies

#### 2.3.1 Search electronic databases

The trials published will be carried out in electronic databases from inception to the March 31, 2020, which consist of PUBMED, EMBASE, Cochrane Library, Scopus, Web of Science, CINAHL, AMED, WANGFANG, VIP, and CNKI. We will not apply any limitations to the language and publication status. The detailed search strategy for PUBMED is presented in Table 1. Identical search strategies for other electronic databases will also be created.

**Table 1:**
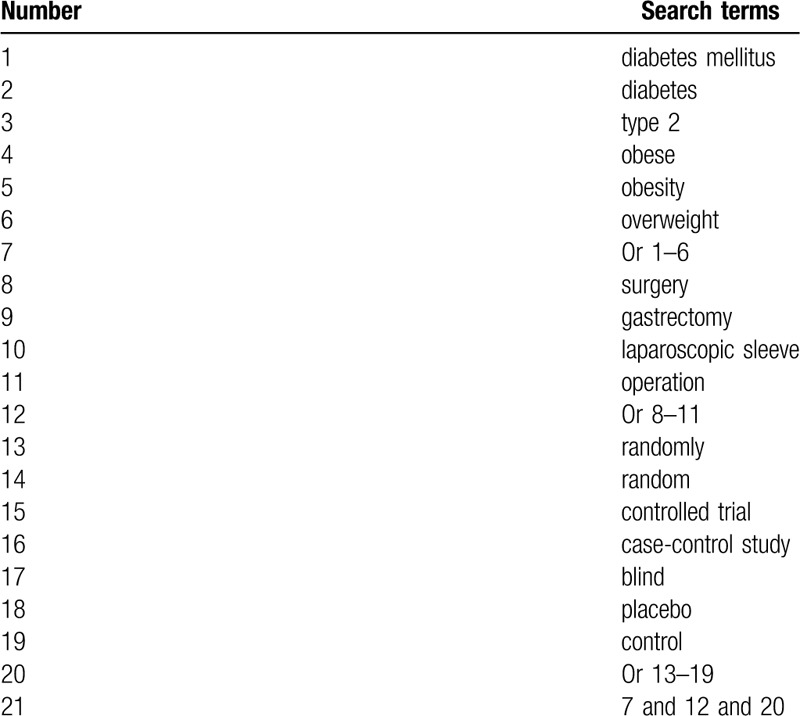
Search strategy applied in PUBMED.

#### 2.3.2 Search for other resources

Besides, we will search other sources to avoid missing any potential trials, such as conference abstracts, dissertations, and reference lists of related reviews.

### 2.4 Study selection and data extraction

#### 2.4.1 Study selection

All identified literatures will be imported to the citation management software (EndNote 7.0), and duplicates will be excluded. Two authors will independently select studies by screening the titles/abstracts of all records based on the inclusion criteria, and non-clinical trials will be removed. Potential eligible trials will be read by full-text to further check if they meet all eligible criteria. In the case of divergences between 2 authors, we will invite a third author to judge and to make a final decision. We will demonstrate the whole process in a flow diagram. All excluded records will be noted with reasons.

#### 2.4.2 Data extraction and management

Two authors will independently perform data extraction according to the predefined standard data extraction sheet. If any different opinions occur between 2 authors, we will ask for a third author help to make a consistent decision. The extracted information comprises of study basic information (such as title, authors, year of publication), study population, sample size, eligibility criteria, diagnostic criteria, randomization, blind, interventions, comparators, outcomes, safety, follow-up information, funding, conflict of interest and any other relevant details.

#### 2.4.3 Dealing with missing data

If we identify any insufficient or missing information, we will request them from primary authors by email. If we can not receive such information, we will only perform obtainable data, and will discuss its potential impacts to the conclusions.

#### 2.4.4 Risk of bias in included trials

Risk of bias assessment will be evaluated by two independent authors using Cochrane Risk of Bias Tool for RCTs, which comprises of 7 aspects. Three different grades (high risk of bias, unclear risk of bias, and low risk of bias) will be utilized to check each aspect for all included RCTs. Any discrepancies between two authors will be resolved by a third author through discussion.

### 2.5 Statistical analysis

#### 2.5.1 Data synthesis

We will apply RevMan 5.3 software to carry out statistical analysis. Dichotomous variables such as incidence of complications will be presented as risk ratio and 95% confidence intervals (CIs). Continuous variables such as complete remission of T2DM, body mass index, partial remission of T2DM, lipids, high sensitivity C-reactive protein, and quality of life will be calculated as mean difference or standardized mean difference and 95% CIs. Heterogeneity will be checked using *I*^2^ statistic test. *I*^2^ ≤ 50% will be regarded as low heterogeneity, and a fixed-effect model will be applied for data pooling. In addition, when necessary, meta-analysis will be conducted if sufficient data on the similar characteristics of study and participant, interventions, comparators, and outcomes are obtained. *I*^2^ > 50% will be considered as obvious heterogeneity and a random-effect model will be utilized. Additionally, we will undertake subgroup analysis to investigate possible causes for obvious heterogeneity. If there is still obvious heterogeneity after subgroup analysis, we will not pool the data, and a narrative synthesis of findings will be conducted. We will compare each outcome in OP with T2DM between patients who received LSGT and those who did not. In case of missing essential data from included trials, we will try our best to obtain it by contacting primary study authors. When it is not possible, we will discuss its potential impacts of missing data on the results.

#### 2.5.2 Unit of analysis

We will only extract and analyze data from the first study period if cross-over trials are included in this study.

#### 2.5.3 Subgroup analysis

We will run a subgroup analysis according to the different study characteristics, population characteristics, details of interventions and comparators, and outcome measurements.

#### 2.5.4 Sensitivity analysis

We will carry out sensitivity analysis to identify the stability and robustness of the findings by excluding trials with high risk of bias.

#### 2.5.5 Reporting bias

When at least 10 included trials are included, we will plan to perform Funnel plot, Egger linear regression test to find any reporting biases exist.^[24,25]^

#### 2.5.6 Overall quality of evidence

We will utilize the GRADEpro Guideline Development Tool to evaluate the quality of study findings. Two authors will independently appraise the quality of evidence, and any disagreements between 2 authors will be solved by a third author through consultation.

### 2.6 Dissemination and ethics

We will disseminate this study on a peer-reviewed journal or a conference meeting. This study will not need ethic approval, because it will not use individual patient data and privacy.

## 3 Discussion

Presently, with increasing number of studies focusing on the efficacy and complications of LSGT for the management of OP with T2DM, evidence-based medicine literature is very necessary to elaborate its efficacy and safety. In addition, no such study has been published before. Therefore, in this study, we aim to summarize most recent high quality studies to explore the efficacy and efficacy of LSGT for the management of OP with T2DM. This study may yield evidence for reference to both clinician and health-related policy maker.

## Author contributions

**Conceptualization:** De-zhi Ke, Rong-rong Yao, Hao Tian.

**Data curation:** Wei-wei Wei, De-wang Su, Hao Tian.

**Formal analysis:** Wei-wei Wei, Xian-dong Fu, De-zhi Ke, Ke-yan Chen.

**Funding acquisition:** Hao Tian.

**Investigation:** Hao Tian.

**Methodology:** Wei-wei Wei, Xian-dong Fu, De-wang Su, De-zhi Ke, Ke-yan Chen.

**Project administration:** Hao Tian.

**Resources:** Wei-wei Wei, Xian-dong Fu, De-wang Su, De-zhi Ke, Rong-rong Yao, Ke-yan Chen.

**Software:** Wei-wei Wei, Xian-dong Fu, De-wang Su, Rong-rong Yao.

**Supervision:** Hao Tian.

**Validation:** Wei-wei Wei, Xian-dong Fu, Rong-rong Yao, Hao Tian.

**Visualization:** Wei-wei Wei, De-wang Su, De-zhi Ke, Rong-rong Yao, Hao Tian.

**Writing – original draft:** Wei-wei Wei, Xian-dong Fu, De-wang Su, De-zhi Ke, Rong-rong Yao, Ke-yan Chen, Hao Tian.

**Writing – review & editing:** Wei-wei Wei, De-wang Su, De-zhi Ke, Ke-yan Chen, Hao Tian.
